# In This Issue

**DOI:** 10.1002/cfg.461

**Published:** 2005


**Comparative and Functional Genomics**
*Comp Funct Genom* 2005; **6**: 1.
Published online in Wiley InterScience (www.interscience.wiley.com). **DOI:** 10.1002/cfg.461

### In this issue

### Epitope-tagged budding yeast strains

In their paper, **Russell Howson** *et al.* describe the production and use of two collections of epitope-tagged budding yeast strains. These collections have either the tandem affinity purification (TAP) tag, or the green fluorescent protein (GFP) tag, inserted at the C-terminus of almost every budding yeast open reading frame.

### Genomic analyses of transport proteins in *Ralstonia metallidurans*

*Ralstonia metallidurans* is better able to withstand high concentrations of heavy metals than any other well-studied organism.**Torsten Von Rozycki and colleagues** present an identification of *R. metallidurans* genes encoding homologues of established and putative transport proteins.

### Special section of papers from the BioLink SIG Workshop at ISMB 2004

**Lynette Hirschman** *et al.* introduce the special section with a report on this recent meeting of the text mining special interest group (SIG).

**Karin Verspoor** discusses the production of a semantic lexicon for biological language processing. She presents an analysis of the utility of the National Library of Medicine's Unified Medical Language System (UMLS) for the construction of such a lexicon.

**David Milward and colleagues** describe a novel ontology-based interactive system for information extraction from scientific abstracts and present a case study of its performance in extracting cofactors from EMBASE and MEDLINE.

**Inderjeet Mani** *et al.* discuss the problems involved in identifying and tagging protein names in text and their approach to solving these problems by producing protein name tagging guidelines.

**Shipra Dingare and coworkers** present a system for identifying named entities in biomedical abstracts, and report on its performance in the BioCreative and Coling BioNLP comparative evaluations.

**Henk Harkema** *et al.* present Termino, a web service for biomedical term look-up. Termino provides an extensive database of terms and a recogniser service for automatic identification, classification and mark-up of names of biological entities in a submitted text.

### Conference Calendar

A listing of genomics related conferences planned for May to July 2005.

Copyright © 2005 John Wiley & Sons, Ltd.

